# Artificial Sperm: New Horizons in Procreation

**DOI:** 10.5041/RMMJ.10319

**Published:** 2017-10-16

**Authors:** Valentin Shabataev, Raanan Tal

**Affiliations:** 1 Department of Urology, Rambam Health Care Campus, Haifa, Israel; 2 Neuro-Urology Unit, Rambam Health Care Campus, Haifa, Israel

**Keywords:** Artificial sperm, azoospermia, male infertility, spermatogenesis

## Abstract

Azoospermia, the absence of any sperm cells from the ejaculated semen, poses a real challenge to the fertility urologist. While there are options to create happy families for azoospermic couples, such as the use of donor sperm and adoption, most couples still want to have genetically related offspring. Advances in urology, gynecology, and fertility laboratory technologies allow surgical sperm retrieval in azoospermic men and achievement of live births for many, but not all azoospermic couples. At present, there are extensive research efforts in several directions to create new fertility options by creating “artificial sperm cells.” While these new horizons are exciting, there are significant obstacles that must be overcome before such innovative solutions can be offered to azoospermic couples. The present review article defines the problem, describes the theoretical basis for creation of artificial genetically related sperm cells, and provides an update on current successes and challenges in the long tortuous path to achieve the ultimate goal: enabling every azoospermic couple to have their own genetically related offspring. Hopefully, these research efforts will ripen in the foreseeable future, resulting in the ability to create artificial sperm cells and provide such couples with off-the-shelf solutions and fulfilling their desire to parent genetically related healthy babies.

## INTRODUCTION

Most men and women desire parenthood in general, and specifically to be the biological parents of a child who carries their DNA.^1^,^2^ In sexually procreating species, genetic information is transferred from generation to generation only by gametes, and only when there is undisturbed unification of the nuclei of a sperm cell and an ovum.

The present article focuses on male factor infertility. From the male partner perspective, fertilization cannot be achieved unless mature sperm cells are available. In cases of complete absence of any sperm cells from the ejaculate, surgical options exist for the retrieval of sperm cells from the testicles themselves or their adnexa. No other ready-to-use and non-experimental options exist to achieve mature sperm cells.

This article attempts to foresee what the future holds regarding production of artificial sperm cells as a solution for azoospermia. Based on current knowledge and ongoing research, there are several possible future directions to overcome azoospermia and provide azoospermic men with artificial sperm cells carrying their own genetic information and which could be used eventually to enable them to have their own genetically related offspring. The new options discussed here are fascinating, but unfortunately not yet ready to be clinically administered to azoospermic couples.

## BRIEF INTRODUCTORY FACTS ON MALE FERTILITY

In past generations, fertilization could be achieved only sexually, and procreation was possible only for fertile couples. The Practice Committee of the American Society for Reproductive Medicine (ASRM) defined infertility as the inability to achieve a successful pregnancy after 12 months or more of appropriate, timed, unprotected intercourse. Earlier evaluation and treatment may be justified based on medical history and physical findings and are warranted after 6 months for women over the age of 35 years.^3^ It is estimated that infertility affects about 186 million people globally and affects about 15% of couples attempting to achieve pregnancy.^4^,^5^ Louise Brown was the first baby conceived with *in vitro* fertilization (IVF), and her birth on July 25, 1978 marked a new era in which fertilization can be achieved in the lab and not only fertile couples but also infertile couples can procreate and have their own genetic offspring.^6^ Nevertheless, reproductive medicine provides a solution for only 70% of infertile couples, so they can have a baby within 5 years after commencing fertility treatments while using either patients’ own or donor gametes.

When discussing infertility, it must be emphasized that sperm quality plays a major role. While it was once thought that most cases of infertility were related to the female partner, recent worldwide research, including in Israel, shows that about half of the infertility cases are related to the male partner.^4^,^7^ The most basic test assessing male fertility is semen analysis, which examines ejaculate. The test is readily available, non-invasive, and can be performed multiple times. According to contemporary data, the median total number of sperm cells in one ejaculate of a fertile man is 255 million cells, the median percentage of live cells is 79%, and the median percentage of motile sperm cells is 61%.^8^ There are no well-established thresholds for semen parameters to define male infertility. However, in clinical use, the lower fifth percentile values of semen parameters in fertile men are commonly used to define “normal” semen analysis. These lower fifth percentile values in fertile men are: semen volume of 1.5 mL, sperm concentration of 15 million cells per 1 mL, total number of sperm cell of 39 million per 1 mL, total sperm motility of 40%, vitality of 58%, and normal morphology of 4%.^8^ If one or more of these parameters is below these fifth percentile values, the semen quality is considered abnormal.

At the extreme end of male infertility, there are men with a complete absence of spermatozoa in the ejaculate, a condition known as azoospermia. Azoospermia affects about 0.6%–1% of all men and 10%–15% of infertile men.^9^,^10^ Azoospermia is commonly classified as either non-obstructive or obstructive. If the cause of azoospermia is obstruction of the seminal tract, sperm cells can be retrieved in virtually all cases. When treating cases of obstructive azoospermia, male fertility specialist centers with micro-surgical capabilities prefer retrieving sperm via micro-surgical epididymal sperm aspiration (MESA)—aspiration of sperm cells from the epididymal head, proximal to the obstruction point. In non-obstructive azoospermia, spermatogenesis in the seminiferous tubules inside the testes is impaired or even totally absent. In some cases, the etiology of non-obstructive azoospermia can be identified and even successfully corrected, leading to the return of sperm cells to the ejaculate. Even if only few sperm cells are found in the ejaculate, not infrequently they may be used for IVF, achieving a successful normal pregnancy and genetic offspring. For men with non-obstructive azoospermia that cannot be improved, there are surgical options for testicular sperm retrieval. The most advanced type of sperm retrieval surgery is called microsurgical testicular sperm extraction (micro-TESE), in which the testicular tissue is micro-dissected under a surgical microscope and individual testicular seminiferous tubules are harvested and sent to the fertility lab for an attempt to find viable mature sperm cells that can be used for IVF. The Department of Urology and Institute of Reproductive Medicine–Weill Cornell Medical College of Cornell University in New York reported testicular micro-dissection sperm retrieval rate ranges of 44%–72% and pregnancy rate ranges of 29%–50% with micro-TESE.^9^

At present, if all measures to retrieve sperm cells (extensive search in the ejaculate, treatment of co-existing conditions that may negatively impact sperm production, and micro-TESE) are not successful, no further options exist to achieve a paternal, genetically related, offspring. In these cases, donor sperm cell use is the only current option for men with non-obstructive azoospermia and failure to retrieve functional sperm cells.

## PARENTHOOD WITHOUT SPERM

For azoospermic couples, there are some options for parenthood that do not require actual maternal ovum fertilization by the paternal sperm cell to achieve a successful pregnancy. Such options include adoption, utilization of sperm donation in cases of failure to produce sperm cells, and embryo donation. Interestingly, embryo donors positively desired donation of their embryos and do not find the decision difficult. Neither donors nor recipients of embryos saw the donation process as akin to adoption.^11^ In addition, research findings imply that neither the lack of a genetic link nor lack of a gestational link between offspring and their parents has a negative impact on parent–child relationships nor adversely impact the psychological well-being of mothers, fathers, or children. Nevertheless, most people strongly prefer having their own genetic offspring.^12^,^13^

## WHAT ARE ARTIFICIAL SPERM CELLS AND HOW MIGHT THEY BE USED?

Spermatogenesis is a biological process in which germ cells (spermatogonia) develop by mitosis, meiosis, and maturation into mature sperm cells (spermatozoa) in the male gonad, i.e. the testis. In production of artificial spermatozoa, genetically related sperm cells are developed from cells that are not essentially germ cells, and the process is performed in the laboratory rather than in the testes. The cells that can be used to create artificial sperm may be either sperm cell progenitor cells or somatic cells.

Intuitively, the immediate potential use of artificial spermatozoa is to replace the non-existent sperm cells in azoospermic men. While at present donor sperm cells are commonly used in fertility interventions in this scenario, once artificial spermatozoa become available for clinical use, they may be used for fertilization and enable the transfer of the genetic information from the father to the developing embryo. Although this technology is not yet ready for use, there is already discussion about the possibilities for using this technology in other circumstances. With artificial sperm derived from cells other than male spermatogonia, single women can use these spermatozoa to fertilize their own oocytes, and same-sex female couples can have an offspring that carries the genetic information from both female partners.^14^ Interestingly, a recently published study shows that while the hypothetical use by lesbian couples is accepted by as many as 68% of the public, the use of artificial sperm by single women wishing to mother a child who carries only their own genetic information without a male-derived genetic component is accepted by only 27%.^15^ Ethically and socially, there are unresolved questions: should the use of artificial sperm be allowed based on technical feasibility, the risk of genetic abnormalities in the offspring, the risk of other adverse offspring-related outcomes, the subject’s personal wishes, society’s acceptance of such intervention, or on other factors?

## WHAT ARE THE OPTIONS TO PRODUCE ARTIFICIAL SPERM CELLS?

Several options to produce artificial sperm cells have been looked at. One of the major obstacles in producing artificial gametes in general and sperm cells in particular is the haploid nature of sperm cells. Being haploid means that these cells contain half the number of autosomal chromosomes and only one sex chromosome, i.e. half of the DNA content in respect to somatic diploid cells. Creating haploid cells mandates strict division of the DNA content of precursor diploid cells. Another option is to use precursor cells that are already haploid; however, this approach narrows the options in choosing precursor cells. The earliest cells in spermatogenesis that are haploid are secondary spermatocytes, cells that have completed the first meiosis. Hence, the only haploid cells in the human body are in the relatively late stages of spermatogenesis, namely secondary spermatocytes, spermatids, and spermatozoa. Of sexually procreating species, haploidy is uncommon. Haploid organisms are mainly organisms of the Hymenoptera order of insects such as ants, bees, and wasps. Of note is that spermatocytes, spermatids, and spermatozoa cannot be found in azoospermic men with complete disruption of spermatogenesis or disruption of spermatogenesis at an early stage. Such cases include Sertoli-cell-only syndrome (germ cell aplasia) and maturation arrest of sperm cells, which commonly occurs at the level of the primary spermatocyte, i.e. a diploid cell that has not yet entered the first meiosis stage.^16^

Using haploid cells poses another challenge, since they do not proliferate in the natural process of spermatogenesis. Several research directions have been explored in the effort to generate artificial sperm cells. The first and possibly most extensively studied option is the use of stem cells—undifferentiated cells with a differentiation potential that may possibly be directed to form gametes in general and specifically artificial sperm cells.^17^ Another interesting direction to achieve ripe spermatozoa is to retrieve spermatids from the testes and to induce, *in vitro*, maturation of spermatids into mature sperm cells. This option sounds very attractive, as only part of the spermatogenic process needs to be artificially reproduced; however, this potential intervention would be an option only in men with late spermatogenetic maturation arrest who can produce spermatids.^17^ Another theoretical option is to direct somatic cells to undergo meiosis, thereby becoming haploid cells.^17^ Generation of artificial sperm cells from somatic cells sounds like a challenging mission, as the differentiation potential of somatic cells is rather limited.

## STEM CELL-DERIVED SPERM CELLS

Stem cells can be found in the embryonic, fetal, and adult stages of life. These cells are undifferentiated and they are able to differentiate and give rise to various tissues and organs. Stem cells are characterized by: (1) an ability to proliferate extensively, (2) clonality (commonly originating from a single cell), and (3) potency (a potential to differentiate and create various types of cells).^18^ Stem cell transplantation into tissues or organs may be a significant and promising step in the management of many incurable diseases. Recently, progress has been achieved in research on stem cell-based therapies in neurology and ophthalmology.^19^,^20^ It has been demonstrated that stem cells can differentiate into various ocular tissues and can potentially be used in the treatment of ocular degenerative conditions and injuries.^20^

There are different types of stem cells; the earlier in development the stem cell, the greater is its differentiation and proliferation potential:^21^

1. Totipotent or omnipotent stem cells—these cells are the most undifferentiated cells, and they are present in the zygote early after fertilization of the oocyte. These cells have the greatest differentiation potential, and every cell can develop into any embryonic or extra-embryonic tissue.
2. Pluripotent stem cells—these cells have a somewhat lower differentiation potential. They can form only intra-embryonic structures, but they are still able to form all tissues and organs since they can differentiate into cells originating from all the three germ layers—ectoderm, endoderm, and mesoderm—which develop. Pluripotent stem cells are present in the inner cell mass of the blastocyst or embryonic germ cells.
3. Multipotent stem cells—these cells are stem cells that can be found in adults, such as mesenchymal stem cells. Mesenchymal stem cells can be derived from various tissues including umbilical cord blood, bone marrow, bone, adipose tissue, Wharton’s jelly, and even from peripheral blood. They have a limited differentiation potential and can give rise only to cells of specific lineage.
4. Unipotent stem cells—these cells are tissue-specific progenitor cells and can give rise to only one specific cell type. Unipotent stem cells include primordial stem cells; in the context of our discussion on artificial sperm cells, the relevant unipotent stem cells are spermatogonial stem cells.

Considering the use of stem cells to generate artificial sperm cells, it must be mentioned that unlike the use of stem cells in regeneration of neural tissues for example, for artificial sperm cells we can only use “patient-specific” stem cells. To transfer the genetic information, the stem cells must be taken from the specific infertile subject and cannot be retrieved from embryos. This requirement limits the possibilities for adult-derived stem cells with a restricted differentiation and proliferative potential. It should be pointed out that all stem cells are diploid.

## USING SPERMATOGONIA OR SPERMATIDS FOR IVF AND *IN VITRO* MATURATION

Spermatids are immature sperm cells. Although they are not yet able to move, they have completed their meiosis and are haploid.

In 1992, the first pregnancy with a single sperm cell directly injected into an oocyte was achieved.^22^ Intracytoplasmic testicular sperm injection (ICSI) opened new possibilities and enabled the use of individual sperm cells, as well as non-motile sperm cells, for IVF. Since then, ICSI has become a popular practice in fertility centers. However, ICSI requires a mature sperm cell, either motile or not. These mature sperm cells may be derived from either the ejaculate or from testicular tissue obtained by testicular biopsy and can be found in about 50% of azoospermic men. In a small subset of azoospermic men who failed micro-TESE surgery, about 0.9%–1%, spermatids in various stages of development could be found.^10^ These cases are classified as late maturation arrest. Direct injection of spermatids into the oocyte has been attempted, and the results correspond with the developmental stage of the spermatocyte: better results with injection of the more mature elongated spermatids but disappointing results with the use of less mature round spermatids. While the reported success rates with elongated spermatids are rather impressive, a 48% fertilization rate and a 29% pregnancy rate, when using round spermatid injections the reported fertilization rate is only about 22% with a 3% pregnancy rate.^10^ Attempts to push the limits further and inject only the nuclei of round spermatids, and even secondary spermatocytes which are even less mature cells, have been reported, but with only anecdotal success.^10^

If the use of round spermatids yields poor results, the next logical step is to develop a technology to induce maturation of these cells *in vitro*. There have been attempts to induce maturation of round spermatids in cell cultures and even of pre-meiotic germ cells, with limited success. Although specific steps such as flagellar (sperm cell tail) growth, nuclear condensation and migration, and development of the acrosome have been observed in cell culture, *in vitro* maturation is a complex process that requires special conditions, and its secrets are yet to be elucidated.

When all maturation steps become feasible *in vitro*, the use of spermatogonia to produce artificial sperm may be a promising option. This option sounds reasonable for azoospermic men who do not produce the more mature forms of sperm cells such as spermatocytes and spermatids, but who do have spermatogonia in the seminiferous tubules. This direction sounds attractive for young males before the onset of puberty who are facing germ cell destruction, such as in the case of pre-pubertal males prior to receiving chemotherapy.^23^,^24^ These young children have spermatogonia that can be derived from the testicular tissue and preserved. After completion of chemotherapy and recovery, this preserved testicular tissue will hopefully be successfully transplanted back to the patient’s testes, repopulating the testes. If spermatogenesis can be restored, they can differentiate *in vivo* into mature sperm cells. This approach is termed fertility restoration. Of course, this approach is not devoid of yet-to-be-discovered theoretical risks, such as the re-implantation of not only spermatogonia but also cancer cells.

## USE OF HUMAN SOMATIC CELLS

As mentioned above, approximately 0.6%–1.0% of men are azoospermic. Of these azoospermic men, pathologic evaluation revealed Sertoli-cell-only syndrome in about 40%–60%.^25^,^26^ Of these, sperm can be retrieved in 44% with micro-TESE.^27^ However, if there are no germ cells in the testis, production of artificial sperm cells from early spermatogonial stem cells or by maturation of more developed cells is not feasible. For patients in whom spermatogonia and/or more developed germ cells are not available, the use of somatic cells to generate artificial sperm cells and serve as a source for genetically related artificial sperm has been considered.^17^,^21^ Several paths have been explored to use somatic cells. It has been shown that somatic cells can behave like germ cells in some sense and express meiotic features and even undergo full meiosis.^17^ The use of somatic cells to induce pluripotent stem cells from somatic cells has also been demonstrated to be feasible.^21^ In this process, pluripotent stem cells with differentiation potential have been created. This acquired pluripotency and the associated acquired differentiation potential can logically be used to create genetically related artificial sperm. Another exciting direction is to use only the nucleus of the somatic cell to transfer the genetic information. In this path, the somatic cells contribute the genetic information contained in their nuclei by transferring the nuclei to spermatogonia derived from a genetically unrelated donor.^21^ The use of somatic cells to produce artificial sperm cells opens new horizons to produce sperm cells even in females, since somatic cells are not gender-specific. In human females, creation of artificial sperm has been reported, but fertilization has not been achieved.^28^

## WHERE DO WE STAND TODAY?

Currently, genetically related artificial sperm cannot yet be used in humans to achieve pregnancy and the live birth of genetically related offspring. Table 1 provides an overview of the current status of efforts toward developing genetically related artificial sperm. Significant scientific achievements have been achieved, with success possible in the foreseeable future.

**Table 1:** The Status of Various Paths for Genetically Related Artificial Sperm Production to Date.^27^

Path	Animal Model			Human		
	Sperm Cell	Fertilization	Offspring	Sperm Cell	Fertilization	Offspring
*In vitro* differentiation of germline stem cells	✓	✓	✓			
*In vitro* proliferation of germline stem cells and auto-transplantation	✓	✓	✓			
*In vitro* differentiation of induced pluripotent stem cells	✓			✓		
*In vitro* differentiation of induced pluripotent stem cells and auto-transplantation	✓	✓	✓			
*In vitro* somatic cell transformation into sperm cells						
*In vivo* somatic cell transformation into sperm cells			✓			

To conclude, directions to create genetically unrelated artificial sperm may theoretically include:^28^

1. *In vitro* differentiation of embryonal stem cells to mature sperm cells.
2. *In vitro* differentiation of embryonal stem cells to progenitor germ cells followed by transplantation into the recipient’s testes and *in vivo* spermatogenesis.

Directions to create genetically related artificial sperm may include:^28^

1. Induction of maturation of spermatogonial cells, either *in vivo* or *in vitro*, for males with spermatogonia and early maturation arrest.
2. Harvesting of testicular tissue before oncological treatment and *in vitro* maturation or self-transplantation and *in vivo* maturation for young males facing an imminent threat to spermatogenesis, such as pre-pubertal children facing chemotherapy.
3. Using somatic cells to induce pluripotent stem cells followed by differentiation to mature sperm cells, or reversed differentiation with induction of meiosis and spermatogenesis in somatic cells, for men with a complete absence of germ cells.

## SUMMARY

There are various ways to achieve parenthood for azoospermic men. Solutions such as sperm donation or adoption are not biologically demanding and readily available. However, couples usually desire their own genetically related offspring. Stem cell research and regenerative biology have suggested several paths to generate artificial sperm for azoospermic men in various clinical scenarios. Thus far, significant steps have been made to manipulate cells, but the ultimate goal of human artificial sperm production is not yet feasible.

## Abbreviations

ICSI: intracytoplasmic testicular sperm injection
IVF: *in vitro* fertilization
MESA: micro-surgical epididymal sperm aspiration
TESE: testicular sperm extraction.
